# An Emergency Medical Services to Emergency Department Checklist for Handoff of Cardiac Arrest: A Modified Delphi Approach

**DOI:** 10.1016/j.acepjo.2025.100071

**Published:** 2025-02-21

**Authors:** Molly McCann-Pineo, Deanna Margius, Devin Howell, Masra Shameem, Timmy Li, Scott Weingart, Jason Groff, Daniel Rolston, Lance Becker, Daniel Jafari

**Affiliations:** 1 Donald and Barbara Zucker School of Medicine, Hempstead, New York, USA; 2 Department of Emergency Medicine, North Shore University Hospital, Manhasset, New York, USA; 3 Department of Emergency Medicine, Nassau University Medical Center, East Meadow, New York, USA; 4 Feinstein Institutes for Medical Research, New York, USA

**Keywords:** cardiac arrest, CPR, EMS, handoff, handover, transition of care, checklist

## Abstract

**Objectives:**

Effective communication during handoffs between emergency medical services (EMS) and emergency department (ED) personnel is a critical step in out-of-hospital cardiac arrest (OHCA) care. No handoff tool has been specifically designed for OHCA, in which timely and accurate transfer of information can substantially affect patient care. This study aimed to develop a standardized checklist for OHCA handoffs based on expert consensus using a modified Delphi approach.

**Methods:**

A panel of experts from EMS and the ED were recruited to rate the importance of 17 preidentified communication items derived from video reviews of OHCA handoffs. Experts completed 2 rounds of identical surveys, followed by participation in a focus group. Participants were asked to rate the importance of each item from 1 to 10 and the timing of when items should be communicated (ie, first, second, or third part of the handoff). The focus group further refined the checklist, finalizing the key elements to be included during a 90-minute virtual session via unanimous consensus.

**Results:**

Eleven experts were approached, 10 completed surveys, and 7 participated in the focus group. The expert panel developed a 13-item checklist (patient age, location/cause of arrest, witnessed arrest, estimated downtime, bystander cardiopulmonary resuscitation, initial rhythm, most recent rhythm, episodes of return of spontaneous circulation, defibrillation attempts, airway type, vascular access, medications administered, and code status). Witnessed arrest, bystander cardiopulmonary resuscitation, and estimated downtime were prioritized for the first part of handoff communication.

**Conclusion:**

This study developed a concise, expert-driven checklist for OHCA handoffs to improve communication between EMS and ED.

**The Bottom Line**

Out-of-hospital cardiac arrest presents unique challenges for emergency medical handoffs due to its urgent, complex nature. Miscommunication during these critical transitions can reduce the ability to provide informed care. This study addresses a gap in standardization for cardiac arrest handoffs, where no specific checklist existed. Using a modified Delphi approach with a panel of experts in emergency and prehospital care, researchers developed a concise, structured checklist of essential data points to be communicated. Items such as patient age, witnessed arrest, and initial rhythm are prioritized for rapid exchange during the initial part of the handoff. This checklist aims to reduce errors, enhance efficiency, and support timely life-saving interventions in emergency settings.

## 1 Introduction

### 1.1 Background

A critical aspect of ensuring continuity and high quality of care in acute care settings is the handoff process, during which patient care responsibilities are transferred from one health care provider to another.^1^ Effective handoffs are particularly vital during high-stakes, high-intensity events, such as cardiac arrest (CA). Communication failures^2^ during these transitions can lead to substantial negative outcomes, making it essential to prioritize effective handoff strategies.

### 1.2 Importance

Although various communication tools to enhance handoffs exist,^3^ none have been specifically tailored to address the unique challenges posed by out-of-hospital CA (OHCA).^4^ The urgency and complexity of OHCA, combined with the necessity for rapid, concise, and comprehensive information exchange, demands a specialized approach. With the incidence of emergency medical services (EMS)-treated OHCA in the United States being 88.8 individuals per 100,000 population, or roughly 300,000 cases annually,^5^ it is critical that all pertinent information be relayed swiftly and in an organized manner to optimize patient care and minimize the risk of information loss during transfer.

### 1.3 Goals of This Investigation

Previous research identified substantial gaps in communication during OHCA handoff.^6^ In response, we aimed to develop a checklist standardizing the handoff process for OHCA. This checklist aims to ensure that all essential information is communicated clearly and concisely, reducing the risk of errors or omissions through the consensus of an expert panel.

## 2 Methods

### 2.1 The Delphi Process

#### 2.1.1 Checklist item development

Based on our institution’s previous work on video review-based studies of CA^6^, ^7^, ^8^, ^9^ and through a preliminary review of CA videos, we identified 17 items or data points most frequently communicated during OHCA handoff between EMS and emergency department (ED) staff.^6^ We then used a modified Delphi approach^10^ to develop a checklist of these items to be utilized during CA handoff.

#### 2.1.2 Selection of experts

The research team purposely identified experts internal and external to our health system in the fields of CA, resuscitation, and prehospital medicine. Potential experts included those from the ED (attending and resident physicians, advanced care providers [ACPs], and nurses) and EMS (emergency medical technicians, paramedics, field training, operations, and leadership), as well as clinician-scientists.

#### 2.1.3 Data collection

Individuals were invited via email to participate in a series of electronic iterative surveys (up to 3) and attend a one-time focus group session. The email stated the study objectives, their expected role, and the process of incorporating their feedback. Those interested were provided with a secure link to a Research Electronic Data Capture (REDCap) survey^11^ containing a consent form and the survey. Participants were asked to rate the importance of each of the 17 items on a scale of 1 = least important to 10 = most important, as well as the timing in which each item should be communicated. Multiple items could receive the same score depending on expert perceptions. In our study of OHCA handoffs treated at our facility, it was previously found that the median handoff time was 66 seconds^6^; therefore, we categorized time of communication into 3 periods to represent the first (0-22 seconds), second (23-44 seconds), and third (45-66 seconds) tertiles of a handoff. Basic demographic information, such as current role and years of experience, was also collected.

#### 2.1.4 Data analyses

After participants completed the first survey, the results were reviewed by the research team. A written summary of the aggregated results was then provided to the participating experts to inform them of the group’s perspectives. They were then asked to complete the survey a second time, approximately 2 months after the first survey. This period was to collect and analyze the data and to obtain the results of the second round. In both surveys, participants were encouraged to submit their suggested additional items for consideration in a final free-text field. Periodic reminders were sent to participants to respond to the survey. We then conducted a one-time focus group session, where the survey results and checklist items were discussed among the expert panel. The focus group was conducted virtually via the secure platform for ease of scheduling and was moderated by authors DJ and assisted by MM-P. Author MM-P has received formal training in qualitative research methods and has experience in conducting focus groups. Study team members and participants may have known one another. The focus group was audio and video recorded for analytical purposes and lasted approximately 90 minutes. MM-P took field notes. The focus group was structured by first presenting aggregate findings from the 2 electronic surveys, followed by an open discussion about all 17 items checklist items. Participants were asked if each item should be included in the final checklist or not, as well as if any other data points were suggested by participants in the previous 2 survey rounds. Any disagreements were discussed, and a formal vote of consensus among all panel members was obtained for the finalized checklist.

#### 2.1.5 Ethical approval

This study was approved by our health system’s Institutional Review Board.

## 3 Results

Eleven experts were identified, with 1 declining to complete the 2 rounds of surveys, resulting in a final sample of 10 experts. Two senior nurses, 1 ACP, 1 emergency medicine resident, 1 emergency medicine attending physician, and 1 clinician-scientist from the ED, along with 1 paramedic, 1 EMS field supervisor, and 2 individuals from EMS leadership (medical and operations), participated. The timeframe of the surveys and the focus group meeting was from December 2023 to March 2024. The median years of experience of the 10 participants were 11 years (range, 2-35) (Table 1). Participants identified witnessed arrest, estimated downtime, and code status as the most important items to include on the checklist (≥70% of responses). Ratings of each item, as well as consensus (percentage of agreements between participants in each round of the survey), are presented in Table 2.

**Table 1:** Expert panel roles and years of experience.

Specialty	Role	No. of participants	Years of experience (range)
ED	ED attending	2	15-20
	ED resident/ACP	3	2-11
	ED nurse	2	4-6
EMS	Field supervisor	2	20-35
	EMT/paramedic	1	10

ACP, advanced care provider; ED, emergency department; EMS, emergency medical services.

**Table 2:** Rating of data points from 1 (least important) to 10 (most important), and percentage of the same response (consensus).

Survey item	First survey	Second survey	First survey	Second survey
	Median item rating (IQR)	Median item rating (IQR)	% Consensus	% Consensus
Location of arrest	6 (4-8)	5 (5-7)	20	50
Witnessed arrest	10 (9-10)	10 (8-10)	70	60
Estimated downtime	10 (10-10)	10 (10-10)	80	80
Bystander CPR	9.5 (9-10)	10 (10-10)	50	80
Patient age	9.5 (7-10)	7.5 (5-9)	50	20
Initial rhythm	9 (8-10)	10 (8-10)	50	60
Most recent rhythm	9.5 (8-10)	9.5 (8-10)	50	50
Episodes of ROSC	9 (7-10)	8 (7-10)	30	30
Time of last ROSC	9 (8-9)	9 (8-10)	40	40
Defibrillation attempts	9.5 (7-10)	8 (8-9)	50	40
Time since last defibrillation	9.5 (7-10)	8.5 (8-9)	50	30
Airway type	7.5 (7-10)	8.5 (7-10)	40	40
RSI use	6.5 (3-9)	6 (5-9)	20	40
ETCO_2_	9 (9-10)	9 (7-10)	44	40
Vascular access	6 (5-9)	8 (6-10)	30	30
Medications administered	8 (6-10)	9 (7-10)	40	40
Code status	10 (8-10)	10 (10-10)	70	80

CPR, cardiopulmonary resuscitation; ETCO_2_, end-tidal carbon dioxide; IQR, interquartile range; ROSC, return of spontaneous circulation; RSI, rapid sequence induction.

Patient age, witnessed arrest, bystander cardiopulmonary resuscitation (CPR), estimated downtime, initial rhythm, and code status were chosen to be communicated in the first tertile of handoff by at least 50% of experts. The time of last return of spontaneous circulation (ROSC), defibrillation attempts, and time of last defibrillation were reported as items to be communicated in the second tertile. In the last tertile, vascular access, rapid sequence induction (RSI) use, end-tidal carbon dioxide (ETCO_2_), and airway type were chosen. Items identified in each of the tertiles did not change across surveys (Table 3).

**Table 3:** Expert panel’s final checklist after focus group meeting and proposed mnemonic.

Expert panel’s checklist	Proposed mnemonic: CARDIAC CPR
Patient age	Cause/location of arrest
Location/cause of arrest	Age
Witnessed arrest	Rhythm (initial and most recent)
Estimated downtime	Downtime (estimated)
Bystander CPR	Interventions (bystander CPR, defibrillation attempts)
Initial rhythm	Airway type
Most recent rhythm	Circulation (episodes of ROSC, vascular access)
Episodes of ROSC	
Defibrillation attempts	Code status
Airway type	Prehospital medications
Vascular access	Report (witnessed arrest)
Medications administered	
Code status	

The left section represents the data elements with no specific order. The right section represents the proposed mnemonic by the authors.

CPR, cardiopulmonary resuscitation; ROSC, return of spontaneous circulation.

Seven out of 10 of the experts participated in the focus group session, whereas 3 declined because of scheduling conflicts despite multiple attempts by the research team to accommodate everyone. After discussion, 13 items were finalized for checklist inclusion by the expert panel (Table 3). The location of arrest was recategorized as “Location/cause, if known” per expert suggestion. The number and time since the last defibrillation were combined into 1 item, “Defibrillation attempts.” Similarly, the number and time since the last ROSC were also combined into 1 item, “ROSC Episodes.” RSI use and ETCO_2_ were agreed on by experts not to be included in the final checklist.

## 4 Limitations

Our work has several limitations. First, although we aimed to include a diverse group of experts, our group was geographically limited and comprised medical staff from academic medical centers within the New York City area. Only 1 expert was recruited outside of our health system; however, we have the largest health system in the state, serving millions of patients annually. Second, our checklist and overall clinical perceptions of advanced cardiac life support (ACLS) may be different from those created in a resource-limited environment with no access to extracorporeal cardiac life support (ECLS), dual defibrillation, or other nonstandard ACLS approaches. Nonetheless, important aspects of the checklist are universal and essential for prognostic and diagnostic purposes. Third, our initial suggested set of items was chosen based on our own experience of video review of OHCA handoff. Finally, scheduling conflicts resulted in a 2-month gap between the surveys and the focus group session, which may have influenced participant responses. This delay was unavoidable as it allowed for maximum panelist participation. Although we believe the delay did not significantly impact their perspectives or the overall results, it did prevent 1 initial participant from attending the final focus group.

## 5 Discussion

In this study, we developed an expert-driven handoff checklist of OHCA from EMS to the ED utilizing a modified Delphi approach. The final handoff checklist includes 13 data points, reduced from the original 17 suggested to the expert panel by the survey. Previous literature suggests operational and cognitive barriers as impediments to effective handoff,^12^ although others identified the need for structured handoff tools.^3^ The process of handoff can be improved using structured checklists.

This OHCA handoff checklist has several advantages: it is structured, concise, and clear. The structured handoff aims to identify key information to increase overall efficiency. One such handoff tool in non-CA patients showed a significantly shorter duration of handoff.^13^ A similarly adapted handoff for OHCA could potentially reduce the time to intervention. The items were chosen based on the ease and clarity of communication by the expert panel, demonstrated by modifications to the location of arrest, defibrillation, and ROSC items, as well as the removal of RSI medications and ETCO_2_. Using a mnemonic that is easy to remember, such as “CARDIAC CPR,” as proposed in Table 3, may further enhance education and improve recall.

Prompt rhythm determination is crucial to the timely implementation of early therapies. Factors such as witnessed arrest, bystander CPR, estimated downtime, and initial rhythm could shift an ED physician’s clinical attention in CA patients amenable to defibrillation toward more advanced therapies. Trials of ECLS and dual sequential external defibrillation and vector change have shown great promise for improved OHCA outcomes, providing early recognition of refractory shockable rhythm.^14^^,^^15^ It is conceivable that a structured handoff, clearly communicating these items, could expedite care and ultimately affect outcomes.

To balance accuracy with efficiency, some items were rated lower in importance or placed in the last tertile of the order of communication. Although these items are important, many of these factors (vascular access and airway type) are visible to the team and, therefore, may be deemed redundant. On the other hand, discussion of pertinent negatives, including lack of definitive airway or vascular access, could mobilize team members to focus their efforts on completing these critical tasks. Furthermore, the expert panelists argued that RSI use and ETCO_2_ do not meaningfully change immediate clinical management. The literature has previously demonstrated the limitations of ETCO_2_ in prognostication,^16^ which was echoed by the panelists during the discussion. Given that most CA patients are intubated without RSI,^17^ the panelists opted to remove this item from the checklist.

Our focus group rated data points in tertiles of importance to be communicated from first to third. It remains to be seen if this order is critical, and a future study could randomize this order and investigate provider perceptions and outcomes. The expert panel did not agree about the tertile location of arrest, most recent rhythm, episodes of ROSC, and medications administered. This could partly be explained by the diversity of panelists’ perspectives with different clinical backgrounds and roles.

In conclusion, we developed an OHCA handoff checklist using a modified Delphi method that aims to deliver the most essential data points. In the future, we will implement this checklist within our ED, and if it is shown to improve communication, we aim to extend it to our entire health system to examine its effects on patient outcomes.

## Author Contributions

MCP: conceptualization, data collection, data analysis, critical review and evaluation of results, primary authorship of the paper, review and editing, study supervision. DM: data collection, data analysis, critical review and evaluation of results, review and editing. DH, DR, LB: conceptualization, critical review and evaluation of results, review and editing. MS, SW, JG: review and editing. TL: conceptualization, data collection, data analysis, critical review and evaluation of results, review and editing, study supervision. DJ: conceptualization, data collection, data analysis, critical review and evaluation of results, review and editing of the paper, study supervision, procurement of grant or other funding.

## Funding and Support

Zoll Foundation. All views expressed in the submitted article are our own and do not represent the official position of the institution or funder.

## Conflict of Interest

All authors have affirmed they have no conflicts of interest to declare.
